# Ethical leadership, corporate social responsibility, firm reputation, and firm performance: A serial mediation model

**DOI:** 10.1016/j.heliyon.2021.e06809

**Published:** 2021-04-15

**Authors:** Nguyen Thi Thao Nguyen, Nguyen Phong Nguyen, Tu Thanh Hoai

**Affiliations:** a CFVG, University of Economics Ho Chi Minh City, Ho Chi Minh City, Viet Nam; b School of Accounting, University of Economics Ho Chi Minh City, Ho Chi Minh City, Viet Nam

**Keywords:** Ethical leadership, Corporate social responsibility, Firm reputation, Firm performance, Vietnam

## Abstract

Due to globalization expansion, corporate social responsibility (CSR) is no longer an unfamiliar concept in emerging markets. In the case of Vietnam, its implementation will be influenced by several factors, including ethical leadership. Drawing upon the stakeholder theory, this study develops and tests a serial mediation model to explain how CSR and firm reputation can connect ethical leadership to enhanced firm performance. The PLS-SEM results from survey data collected from 653 mid- and top-level managers from large companies in Vietnam indicate that ethical leadership positively influences CSR, which, in turn, results in enhanced firm reputation and firm performance. This study contributes to research on the intersection between CSR and leadership in the context of emerging markets. This study also provides some managerial implications for Vietnamese firms striving to promote ethical leadership to achieve CSR outcomes.

## 1 Introduction

Ethical leadership refers to the values and acts of leadership that conform to ethical norms. It can be described as the display of proper social behavior through personal acts and interpersonal relationships and the encouragement of such conduct to followers in a two-way contact process (Brown et al., 2005). The performance implications of ethical leadership have been widely examined in previous studies in Western countries (e.g. Angus-Leppan et al., 2010; Neubert et al., 2009; Shin et al., 2015; Eisenbeiss et al., 2015; Wang et al., 2017), which have suggested different pathways that link ethical leadership to firm performance. For example, Shin et al. (2015) claimed that ethical leadership enhances firm performance by promoting firm-level ethical and procedural justice climates. Another study by Wang et al. (2017) reported that ethical leadership contains humane leadership orientation, leadership accountability and sustainability orientation, which are conducive to organizational success.

Corporate social responsibility (CSR) is defined as a philosophy whereby businesses voluntarily incorporate social and environmental, ethical and human rights issues in their business activities and relationships with their stakeholders (EC, 2011). Our review of the literature found that the connection between ethical leadership and CSR, as well as the focus of scholars on this topic, has been well documented (Saha et al., 2020). However, how ethical leadership can become a firm's reputation and performance through CSR mechanisms has not been investigated, particularly in emerging markets where ethical issues continue to be a major concern. This gap must be bridged to provide significant implications for firms across emerging markets to initiate ethical leadership to maximise the impact of CSR activities towards enhancing firm effectiveness.

In response, we conducted our study in Vietnam, which is an increasingly important emerging market that has seen rapid economic growth and foreign investment. Vietnam represents an appropriate context for CSR as Vietnamese businesses are familiar with the concept. The benefits of CSR are diverse and significant for corporate governance and future development. CSR is a tool that enables businesses to establish a positive corporate image both externally and internally, which maximises profit and enhances their competitive advantage. In Vietnamese business practice, corporations previously had to implement CSR because of exporting requirements from Western companies. However, many Vietnamese companies now realize that domestic customers are becoming more concerned about corporations’ responsibilities and that CSR could make their business more sustainable and provide them with more competitive advantages. On the other hand, Vietnamese businesses are under pressure because the country is gradually losing its competitive edge; therefore, they urgently need to find another advantage to strengthen their position and access more global opportunities.

However, Vietnamese businesses are not actively involved in sustainable growth, as current corporate operations are still motivated by profitability (Vuong et al., 2021). Moreover, the lack of practicality in the current studies, in addition to the lack of studies in this field in Vietnam, indicate an immediate demand for more rigorous research to which companies and CSR practitioners can refer. As we aim to explore the moderated mediation model between ethics, CSR, firm credibility and firm results, this study is important for CSR advocates.

This study will investigate whether two important areas of ethical leadership and CSR are interwoven to foster firm reputation and firm performance in Vietnam. Firm reputation is the public's impression and evaluation of how a company conducts its business (Gotsi and Wilson, 2001). Firm performance should be measured organizationally in terms of both financial and operational efficiency (Venkatraman and Ramanujam, 1986a, Venkatraman and Ramanujam, 1986b). The research question focuses on whether CSR and firm reputation serially mediate the effect of ethical leadership and firm performance. The rationale of the mediating effects is as follows: the employees' views of the environment of their work unit, including CSR values, may be affected by whether they consider the leader to be ethical (Choi et al., 2015). An ethical working environment would promote employee engagement and assist ethical leaders with corporate decision-making. All these outcomes can impact a company's reputation and bottom-line performance.

The rest of this paper is structured as follows: the theoretical background and research model with the corresponding hypotheses are provided in the next section; the data collection, design and sampling methods are then explained; the results of the research are then discussed; and the study concludes with the theoretical and managerial implications and conclusions of the papers.

## 2 Theoretical background and hypothesis development

### 2.1 Ethical leadership and CSR

Ethics refers to a set of rules and principles that guide humans' conduct (Johnstone and Crock, 2012). Ethical behavior could be simply defined as a strong connection between the concepts of right, proper, and fair (Hosmer, 1987). On the other hand, a community's ethical behavior is generally shaped by each country's legal and regulatory systems (Harrison, 1988). The connection between ethics and leadership is naturally created because it is often considered a significant component of gaining happiness and blessing (Levine and Boaks, 2014). Accordingly, associated with ethics, ethical leadership refers to an individual who models behaviors that significantly influence groups or group members to encourage and guide them toward achieving a common goal (Mendonca and Kanungo, 2006). They are conscientious persons who follow on beliefs and ideologies and include the capabilities to contribute to society's growth (Maak and Pless, 2006). According to Bowie (2000), ethical leaders should never evaluate their people only by their contributions and personality. Ethical leadership is characterized as modeling definitionally appropriate behavior through individual acts and interpersonal relations and encouraging team members' behavior through two-way dialogue, support and guidance, and decision-making (Brown et al., 2005). Definitionally appropriate behavior represents the moral aspect of ethical leaders who embody admirable qualities such as integrity, reliability, and trustworthiness by taking responsibility for their decisions, including using a sufficient incentive and penalty system to encourage ethical behaviors and eliminate unethical behaviors; and also to discipline employees responsible for their actions (Piccolo et al., 2010; Brown et al., 2005). Even though ethics has not always been considered an essential characteristic of leadership, this perception gradually changes. Ethic leadership is becoming a distinct leadership style, different from other leadership styles, such as spiritual leadership, authentic leadership, and transformational leadership. Ethical leadership can also be used as an analytical tool to explain certain behaviors' effectiveness and predict what behaviors will be effective in certain contexts (Brown et al., 2005).

Based on conceptual explanations of ethical leadership, it is proven that a connection between ethical leadership and CSR exists in terms of business ethics and corporate growth. According to Ciulla (2005) commented on ethical leadership; it is ethical by being effective. If change is required and necessary, the achievement of the appropriate outcome is ethical at that time.

According to Prout (2006), social responsibility is defined by the capacity to grow stakeholder's wealth, increase workers performance productivity, enhance living standards as well as share the earned values with local society. Similarly, CSR is defined as a philosophy that integrates company activities, activities and relationships between stakeholders (EC, 2011). According to Freeman's stakeholder theory (1988), if an organization devotes its energy to stakeholders' issues, all individuals who are influenced by the organization's decisions, then the profit will be made. CSR could be simply explained as the companies' critical conduct and responsibility for local performing business's relative consequences. Beyond the profit, a CSR-engaged company will consider both the favorable and unfavorable implications of three key elements, including society, environment, and economy (Marsden, 2001). These definitions indeed illustrate the close relationship between CSR and corporate management. In the stakeholder theory, leadership's role in serving its stakeholders' interests supports the connection between leadership and CSR.

According to Hind et al. (2009), CSR initiatives are regularly approached in the wrong way or driven into PR and window dressing. There are no effective CSR implementations without leadership support at all levels (Knippenberg and de Jong, 2010). Simultaneously, ethical leaders will seek to strengthen their environmental, social, and ethical efficiency to fulfill stakeholders' needs. The response of business leaders to CSR initiatives may be influenced by stakeholders' evolution regarding CSR (Morsing and Schultz, 2006). In practice, numerous criminal cases involving corporations show that, even when CSR is working well, they may still commit criminal acts that violate the business's ethics. In such cases, the cause was not always misconceptions about CSR but was the negligence of unethical leaders. In contrast, ethical leadership helps followers care about stakeholders' interests beyond their own, and fosters a sense of corporate social responsibility (Choi et al., 2015). Ethical leadership also emphasizes transparency, accountability and dignity, as well as a desire to do what is right and beneficial to CSR activities (Angus-Leppan et al., 2010). In light of the above reasonings, we propose the following hypothesis.

Hypothesis 1 (H1) Ethical leadership has a positive effect on CSR.

### 2.2 CSR and firm reputation

Firm reputation is defined as the overall perceptual characteristics drawn from its history and attributed to the firm as a predictable indicator of its future performance compared with its competitors (Lange et al., 2011; Walker, 2010). Firm reputation is perceived as a valuable immaterial property that can be strengthened or ruined by the organizational decision to participate or ignore CSR practices and disclosures (Shim and Yang, 2016). Firm reputation reflects two essential intangible assets which can differentiate firm from their rivals. The first one is the impossibility to imitate. The second one is the capability to attract more assets and people (Surroca et al., 2010). Furthermore, corporate reputation has also been described as a pledge of the firm's capacity to fulfill stakeholders' interests (Beheshtifar and Korouki, 2013), which is similar to the aim of CSR implementations.

On the other hand, as Shapiro (1983) stated, the firm's reputation is as healthy as its unique products and services. In addition to the quality of the firm's offerings, CSR is considered to be a significant factor in shaping a firm's reputation (Worcester, 2009). CSR-engaged businesses can enhance their image in the local community, contributing to enhancing the competitive position in their business sectors (Jorge et al., 2015).

The firm's CSR engagement level also parallels employee commitment, strengthening the firm's reputation (Stawiski et al., 2010). This relationship has motivated many researchers to clarify the positive relevance of different perspectives (Lai et al., 2010; Stanaland et al., 2011). Some research found a positive relationship in customers' eyes (Lai et al., 2010; Stanaland et al., 2011), while others are from employees' perspectives (Stawiski et al., 2010). Based on the evidence mentioned, the following hypothesis can be formed.

Hypothesis 2 (H2) CSR has a positive effect on firm reputation.

### 2.3 Firm reputation and firm performance

According to economist Milton Friedman, business's primary purpose is to maximize profits while serving its owners and stakeholders (e.g., shareholders, employees, and customers). Therefore, guaranteeing financial output is the most practical measurement for corporate performance. Corporate image can increase customers' expenditure and make them more loyal to the corporation (Bhattacharya and Sen, 2003), positively affecting corporate performance. In the long-term development journey, increased firm reputation leads to more extraordinary business results (Chong, 2009). Firm credibility is a dependable indicator of customer satisfaction and customer retention (Nguyen and Leblanc, 2001). Likewise, Awang and Jusoff (2009) also found that firm reputation significantly affects establishing a competitive advantage that differentiates it from its competitors.

Consequently, firm reputation will gradually be enhanced, followed by an increase in sales volume, a competitive advantage, and, finally, a higher level of firm performance. Besides, supporting research shows the decisive relevance of firm reputation and firm performance, both financially and non-financially (Black et al., 2000; Pham and Tran, 2020; Sabate and Puente, 2003). Under such circumstances, the following hypothesis can be established.

Hypothesis 3 (H3) Firm reputation has a positive effect on firm performance.

### 2.4 CSR and firm performance

Behaving unethically is a crucial problem because it can be financially detrimental to the entire organization (Collins, 2009; Jensen, 2017). This statement means that ignoring business ethics can negatively affect company performance. As stated by Garriga and Melé (2013), throughout the history of developmental economics, CSR has been seen as vital to achieving economic goals and prosperity. CSR involvement in business enhances firms' values, even in controversial industries (Cai et al., 2012). The relationship between the two variables was even stronger in sensitive industries (Reverte, 2012). In the direct approach, it has been argued that CSR decreases firms' cost of equity (Chava and Purnanandam, 2010; Dhaliwal et al., 2011; El Ghoul et al., 2011) and cost of debt (Chava and Purnanandam, 2010; Goss and Roberts, 2011). Environmental responsible initiatives can reduce operating costs, leading to improved financial results (DiSegni et al., 2015). In addition to waste minimization, sustainable corporate growth has a significant effect on productivity. It is closely linked with CSR stakeholders' satisfaction (Khaleel et al., 2017). CSR-engaged businesses strategically keep improving their image and build outstanding ties with shareholders that effectively improve their business performance (McWilliams and Siegel, 2001; Turban and Greening, 1997; Davis, 1973). The longer-lasting social-organizational relationship built from CSR activities encourages the customers’ willingness to purchase goods and services offered by companies engaging in CSR (Dobers and Halme, 2009). Higher firm reputation leads to higher financial performance (Roberts and Dowling, 2002; Suresh et al., 2001).

In the indirect approach, CSR has a strong influence on employees' involvement and corporate risk management. CSR activities' engagement helps establish a stable association between employees and the company that significantly raises staff loyalty and increases firm performance progressively (De Roeck et al., 2014). The CSR-implemented companies purposely progress their workforce to satisfy their desire for long-term sustainable growth (Zhu et al., 2014). First, CSR bonds the affiliation between employees and corporations, which encourages increased employee productivity and commitment and, as a result, also increases corporate performance (Zhou, 2016). Furthermore, CSR involvement in corporate strategy is advantageous in attracting talent (Lim and Greenwood, 2017). Second, CSR activities are mostly published (Dhaliwal et al., 2011); consequently, they increase corporate transparency. With greater transparency in stakeholder communication, there is greater control in firm risk management. The substantial risk from various financial, social, and environmental crises can be reduced to secure the firm's cash flow (Sharfman and Fernando, 2008). Several papers have followed the line of sustained argumentation for the relationship between CSR and firm performance (e.g., Alafi and Al Sufy, 2012; Galbreath and Shum, 2012; Luo and Bhattacharya, 2006; Margolis and Walsh, 2003; Pham and Tran, 2020; Rehman et al., 2020; Rowley and Berman, 2000). All available evidence supports the following hypothesis.

Hypothesis 4 (H4) CSR has a positive effect on firm performance.

Hypothesis 5 (H5) CSR and firm reputation serially mediate the relationship between ethical leadership and firm performance.

**Figure 1:**
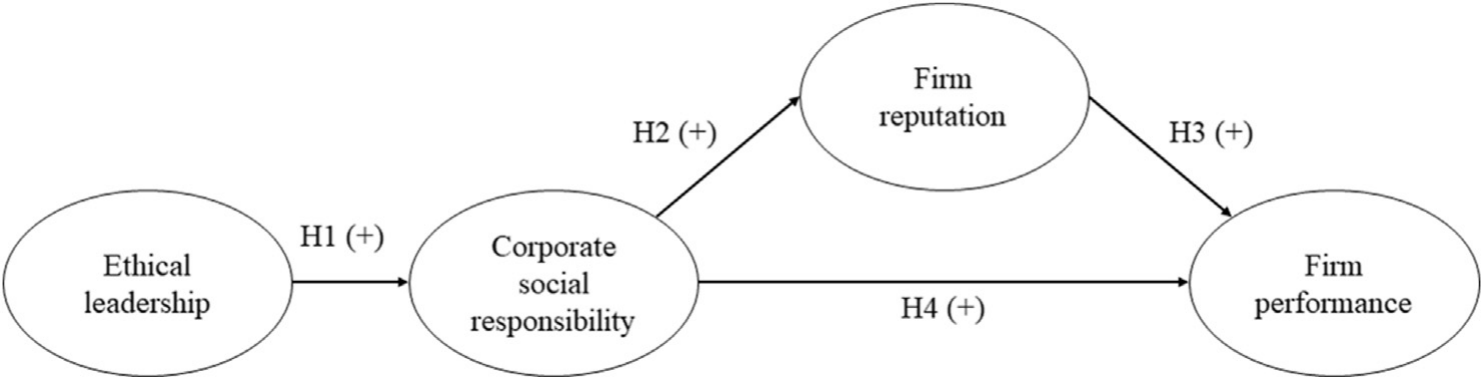
The proposed model and hypotheses.

The proposed model and corresponding hypotheses are shown in Figure 1.

## 3 Research methodology

In this study, the scope of data collection was limited to two industries: manufacturing and services. Furthermore, because of the media visibility of CSR operations in large enterprises, the sample was limited to large enterprises (Graafland et al., 2003). The larger the business, the more money is devoted to corporate responsibility activities compared to small and medium enterprises (Fifka and Pobizhan, 2014). As defined in the Vietnamese Government's Decree 56 ND-CP, a large manufacturing enterprise is required to have more than VND 100 billion in its capital account or more than 300 permanent employees. The capital required for the service sector amounts to more than VND 50 billion or over 300 full-time workers.

In this study, the target respondents were mid- and top-level managers of qualified firms because of their significant role in their strategic growth (Hansen et al., 2008). According to previous studies (e.g., Hansen et al., 2008; Wei et al., 2019), these managers represent their companies in answering survey questionnaires. Moreover, the managerial levels would be fully aware of the performance outcomes of organizational strategies. A complex and systematic approach determined potential respondents. Using LinkedIn, the globally professional networking website (Mintz and Currim, 2013), the first sampling frame was built. Collecting professional emails through LinkedIn is common in academic papers (e.g., Mintz and Currim, 2013; Ouakouak and Ouedraogo, 2017). Despite this approach's time-consuming nature, numerous advantages make it worthwhile: cost-saving, rapidity of recruiting, approachability to the information source, and the possibility to access a diversified sample (Stokes et al., 2019). LinkedIn provides the possibility to approach potential respondents living all over Vietnam. Therefore, the sampling scope was composed of professionals who have visible details on their LinkedIn profile, such as the workplace, email address, and management job titles. The survey had been approved by the Research Ethics Committee of the University of Economics Ho Chi Minh City (Vietnam). All participants provided informed consent at the commencement of the survey, and all their responses remained completely anonymous.

Of the 9,940 LinkedIn users on the invitation list, 1,428 respondents shared their opinions through an online survey. The eligibility of respondents and the completeness of survey forms were all double-checked. After examining the validation of responses, 418 incomplete answers, 193 answers from trading companies, and 164 from small companies were discarded. A further verification via email was done for the remaining 653 profiles. The number of verified respondents who had used their email was much higher than those who had used their company one, 551 and 102, respectively. Subsequently, a callback was conducted by directly calling or emailing 100 (or 18%) personal-email users to confirm their affiliation with the company. Only 13 out of the 100 callbacks were no longer attached to the company disclosed on their LinkedIn profile, which resulted from job switching. However, it is confirmed that their responses relied on their practical experiences in the former company. Acknowledging that the unit of analysis is at the firm scale, a strategic scanning process was taken on multiple responses to prevent duplicate responses from the same organization. Following Brislin (1970), the English version questionnaire was initially written. Then the forward and backward translation method was applied to convert it into a Vietnamese version. The translation process was performed by two language professionals who are proficient in both English and Vietnamese. The translation was then proofread and rechecked by managers and academics before distributed to potential participants via Survey Monkey, a web-based survey tool.

This study uses well-established scales in the literature to measure the main constructs. The evaluation scales applied in this paper can be seen in Table 2. The scale of ethical leadership is a ten-item scale adopted from Brown et al. (2005), while CSR was assessed by a 17-item scale proposed by Wu et al. (2015). In addition, this study rated corporate reputation using the scale of Fombrun et al. (2000) and Rettab et al. (2009).

It was not easy to find objective firm performance data in Vietnam; therefore, a subjective self-evaluation measure was used. The self-evaluation measure of performance has been widely applied in earlier business ethics studies (e.g., Wei et al., 2019) and marketing (e.g., Hansen et al., 2008). It is argued that there is a robust positive correlation between self-evaluation and objective firm performance measures (Venkatraman and Ramanujam, 1986a, Venkatraman and Ramanujam, 1986b). This study measured firm performance following Fornell (1992) and Morgan and Piercy (1998). Participants were asked to rank their firm performance over the last three years compared to key competitors. There was a different number of response categories for each rating scale. Both firm reputation and firm performance had six items, while ethical leadership and corporate social responsibility were ten and seventeen. Except for the verbal anchor of firm performance, which was from poor to excellent, other designs were from strongly disagree to strongly agree. To diminish common method bias, different scale endpoints were consciously designed (Podsakoff et al., 2003).

As mentioned previously, this survey applied different measures for the various variables referred to as the theoretical framework. The relations between these constructs did not need to be clarified by the respondents. The theoretical relationships between variables, including ethical leadership, CSR, firm reputation, and firm performance, were examined through statistical methods. With this study's context and structure, the survey was effective and convenient, producing higher quality data.

## 4 Research results

### 4.1 Sample characteristics

Table 1 presents the characteristics of the sampled firms. As can be seen in Table 1, the sample is spread across different industry sectors. Of 653 acceptable samples, 62.17% were services firms, and 37.83% were manufacturing firms. These figures reflect the service-oriented trend in the Vietnamese sector structure, in which the services industry accounts for 41% of Vietnam's GDP (Deloitte, 2020). In terms of ownership structure, 70.44% of sample firms were foreign-invested enterprises; 29.56% are businesses without foreign ownership capital. In terms of firm size as measured by the owned asset, 80.77% of examined samples were firms with assets over 200 VND billion, while most were firms with assets over 1,000 VND billion (48.70%) Regarding firm size, as measured by the number of employees, 27.56% of samples were firms with 301–1,000 full-time staff, and 24.66% were firms with 1,001–5,000. In third place is 20.98 percent of companies with 101–300.

**Table 1:** Characteristics of sample firms (n = 653).

Demographics	n	%
**Firm size (assets in VND billion)**		
≤10	14	2.15
11–50	19	2.91
51–100	42	6.43
101–200	51	7.81
201–500	90	13.78
501–1,000	119	18.22
>1,000	318	48.70
**Firm size (employees)**		
≤50	27	4.13
51–100	42	6.43
101–300	137	20.98
301–1,000	180	27.56
1,001–5,000	161	24.66
5,001–10,000	56	8.58
>10,000	50	7.66
**Types of ownership**		
Private company	171	26.19
JV with a local partner	24	3.67
JV with international partner	59	9.03
100% foreign-owned enterprise	302	46.25
SOEs (≥51% states capital)	41	6.28
Others	56	8.58
**Ownership structure**		
With foreign capital	460	70.44
Without foreign capital	193	29.56
**Industry sector**		
Hotel and restaurant	45	6.89
Financial, banking, insurance	112	17.15
Information and communication	56	8.58
Wholesale and retail	18	2.76
Transport and warehouse	23	3.52
Construction	40	6.13
Agriculture, forestry, fisheries, and mining	31	4.75
Processing industry and manufacturing	106	16.23
Health and social aid	21	3.22
Consulting (e.g. accounting, law, and architecture)	47	7.20
Education and training	14	2.14
Arts, entertainment, and recreation	9	1.38
Others	120	18.38
**Industry type**		
Manufacturing	247	37.83
Services	406	62.17

### 4.2 Assessment and refinement of the measurement model

At first, the measurement model was examined for reliability and validity. As shown in Table 2, the outer loadings and the corresponding t-bootstrap values resulted from the test are acceptable and highly reliable. The outer loadings of all observed variables range from 0.59 to 0.92, exceeding the 0.50 limit (Hulland, 2012). Consistent with this, all the equivalent t-bootstrap values fall within the statistical significance range of 19.80 and 125.52, much greater than 1.96. In addition, the convergent validity fulfilled expectations because the average variance extracted (AVE) values for all constructs are over the cutoff value of 0.50, within the range of 0.63 and 0.79. The composite reliabilities (CR) of the latent variables range from 0.95 to 0.97, surpassing the satisfactory standards for exploratory research (Kline, 2016).

**Table 2:** Scale items and latent variable evaluation.

Construct and items	Outer loading	t-value
**Ethical leadership (AVE = 0.67, CR = 0.95)** (Brown et al., 2005)		
• Our senior managers conduct their personal lives in an ethical manner	0.78	40.60
• They define success not just by results but also by the way that they are obtained.	0.78	45.80
• They listen to what employees have to say	0.83	60.58
• They discipline employees who violate ethical standards	0.67	22.96
• They make fair and balanced decisions	0.83	55.65
• Our senior managers can be trusted	0.85	71.05
• They discuss business ethics or values with employees	0.85	67.53
• They set an example of how to do things the right way in terms of ethics	0.83	62.50
• They have the best interests of employees in mind	0.85	63.58
• When making decisions, our senior managers ask, "what is the right thing to do in terms of business ethics?"	0.86	82.59
**Corporate Social Responsibility (AVE = 0.63, CR = 0.97)** (Wu et al., 2015)		
• Our company always pays its taxes on a regular and continuing basis.	0.59	19.80
• Our company complies with legal regulations completely and promptly.	0.70	29.10
• Our company participates to the activities which aim to protect and improve the quality of the natural environment	0.78	37.45
• Our company implements special programs to minimize its negative impact on the natural environment.	0.80	38.35
• Our company makes investments to create a better life for future generations	0.85	62.54
• Our company targets sustainable growth which considers the future generations.	0.86	62.06
• Our company supports non-governmental organizations working in problematic areas.	0.76	36.04
• Our company contributes to the campaigns and projects that promote the well-being of society	0.79	43.50
• Our company protects consumer rights beyond legal requirements.	0.85	63.20
• Our company provides full and accurate information about its products or services to its customers.	0.81	46.55
• Customer satisfaction is highly important for our company	0.74	35.16
• Our company encourages its employees to participate to the voluntary activities	0.75	29.70
• Our company policies encourage the employees to develop their skills and careers	0.80	50.10
• The management of our company primarily concerns with employees' needs and wants	0.85	79.15
• Our company implements flexible policies to provide good work and life balance for its employees.	0.83	67.11
• The managerial decisions related to the employees are usually fair.	0.81	57.62
• Our company supports employees who want to acquire additional education.	0.79	43.76
**Firm reputation (AVE = 0.74, CR = 0.95)** (Fombrun et al., 2000; Rettab et al., 2009)		
• In general, our organization has a good reputation	0.87	59.01
• We are widely acknowledged as a trustworthy organization	0.87	54.08
• This organization is known to sell high-quality products and service	0.89	67.44
• Our company is known to comply with all laws regarding hiring employees and employees benefits	0.88	88.00
• Our salespersons and employees have the reputation of providing full and accurate information to all customers.	0.86	70.44
• Our company is known for giving active support to programs with good social causes.	0.76	40.98
**Firm performance (AVE = 0.79, CR = 0.96),** (Fornell, 1992; Morgan and Piercy, 1998)		
• Market share	0.86	57.15
• Customer satisfaction	0.87	77.86
• Customer retention	0.88	90.87
• Sales growth	0.91	101.17
• Sales revenue	0.92	125.52
• Overall profitability	0.86	58.48

Discriminant validity was assessed using Fornell and Larcker’s (1981) proposed procedure. As stated in Table 3, except for all the control variables, the square roots of AVE of the critical constructs (ranging from 0.79 to 1.00) are all over the equivalent bootstrapped correlation coefficients. Furthermore, the majority of correlation coefficients are determined to be consistently lower than the acceptance value of 0.70, while no single correlations (ranging from -0.10 to 0.85) are larger than their composite reliabilities (ranging from 0.95 to 0.97). These pieces of evidence affirm the eligibility discriminant validity of the main constructs. The Heterotrait–Montrait (HTMT) test was also used (Henseler et al., 2014). The test results in the HTMT values are within the range of 0.03 and 0.89, which is below 1.00, consequently, stronger supporting the discriminant validity (see Table 4).

**Table 3:** Discriminant validity analysis.

	1	2	3	4	5	6	7
1. Ethical leadership	**0.82**						
2. Corporate social responsibility	0.76∗∗	**0.79**					
	*0.79*						
3. Firm reputation	0.72∗∗	0.85∗∗	**0.86**				
	*0.76*	*0.89*					
4. Firm performance	0.56∗∗	0.59∗∗	0.67∗∗	**0.89**			
	*0.59*	*0.61*	*0.70*				
5. Ownership	-0.04	-0.08	-0.10	-0.01	**1.00**		
	*0.05*	*0.09*	*0.10*	*0.03*			
6. Assets	0.01	0.05	0.07	0.06	-0.04	**1.00**	
	*0.03*	*0.05*	*0.07*	*0.06*	*-0.04*		
7. Employees	-0.06	0.01	0.06	0.04	-0.03	0.42∗∗	**1.00**
	*0.06*	*0.05*	*0.07*	*0.06*	*0.03*	*0.42*	

Notes: 1^st^ value = correlation between variables (off diagonal); 2^nd^ value (italic) = HTMT ratio; bold diagonal: square root of AVE; ∗, ∗∗: correlation is significant at the 5% and 1% levels, respectively, (two-tailed t-test).

**Table 4:** Hypothesis testing results.

Dependent variable		Model 1	Model 2		Model 3		
		PERF	CSR	PERF	CSR	FR	PERF
*Hypothesis*	*Independent variable*						
H1	EL	0.60	0.76	0.28	0.76		
		(19.72)^c^	(44.15)^c^	(5.28)^c^	(45.26)^c^		
H2, H4	CSR			0.38		0.84	0.09
				(6.92)^c^		(61.50)^c^	(1.60)^a^
H3	FR						0.59
							(10.12)^c^

	*Control variable*						

	Ownership	0.02		0.04			0.06
		(0.58)		(1.13)			(1.99)^c^
	Assets	0.03		0.02			0.01
		(0.82)		(0.55)			(0.40)
Employees	0.06		0.05			0.00
	(1.70)^a^		(1.34)			(0.09)
Adjusted *R*^*2*^of PERF		0.33		0.38		0.45	

	Indirect effect	Estimate	LLCI	ULCI
H5	EL→CSR→FR→PERF	0.29 (7.30)^c^	0.23	0.38

*Notes*: EL: Ethical leadership; CSR: corporate social responsibility; FR: firm reputation; PERF: firm performance; numbers in brackets: *t*-values; a, b, c denote significance at 10%, 5%, and 1% levels respectively (two-tailed *t*-test); LLCI = lower level of the 95% confidence interval; ULCI = upper level of the 95% confidence interval.

### 4.3 Hypothesis testing results

To test the posed hypotheses, three models were run. Model 1 had the direct effect of ethical leadership on firm performance; Model 2 was an augmentation of Model 1 with corporate social responsibility added as the mediating variable; and Model 3 was the full model with corporate social responsibility and firm reputation acting as two mediating variables in the relationship between ethical leadership and firm performance. H1 proposes that ethical leadership (EL) positively affects corporate social responsibility (CSR). Our analysis supports this hypothesis as the correlation between EL and CSR was positive and significant (β = 0.76; t-value = 44.15: Model 2; β = 0.76; t-value = 45.26: Model 3). H2, which conjectures that CSR positively influences firm reputation (FR), was confirmed as the CSR–FR relationship was positive and significant (β = 0.84; t-value = 61.50: Model 3). Our analysis also supports H3, which posits that FR has a positive effect on firm performance (PERF) (β = 0.59; t-value = 10.12: Model 3). H4 on the positive relationship between CSR and PERF was also confirmed (β = 0.38; t-value = 6.92: Model 2). This study also found that the addition of FR as the mediator in the relationship between CSR and PERF results in the insignificance of the CSR–PERF relationship (β = 0.09; t-value = 1.60: Model 3), suggesting that FR fully mediates the CSR–PERF link. To test H5 on the serial mediating effects of CSR and FR in the EL–PERF relationship, this study calculated the indirect impact of CSR and FR on the EL→CSR→FR→PERF path. The result shows that the indirect effect was significant (β = 0.29; t-value = 7.30: Model 3), and the confidence interval of the effect does not contain zero (LLCI = 0.23; ULCI = 0.38), supporting H5.

## 5 Discussion and implications

### 5.1 Theoretical implications

Given that several recent research papers address CSR's role in enhancing sustainable business (e.g., Samy et al., 2010; Sundström et al., 2020), the business environment's complexity and business capacity are inspiring more in-depth multi-dimensional studies. With the intention of expanding the literature of CSR, especially in the context of Vietnam, this study contributes empirical evidence by testing the mediating model of ethical leadership, CSR, firm reputation and firm performance. The findings show that the positive relationship among the four variables supports the hypotheses of the positive connections among previous studies' variables and provides a well-founded premise for further related studies. In doing so, our study responds to a call by Nguyen et al. (2018) for firms in Vietnam to understand how a Western phenomenon, such as CSR, is essential, meaningful and conceptualized for Vietnamese management.

As stated in the findings, the first hypothesis between ethical leadership and CSR was strongly positively clarified. It was determined that ethical leadership is essential to successfully implement CSR. Ethical leadership is believed to have a strong sense of CSR awareness and willingness to implement CSR; therefore, it results in high-quality CSR management and initiatives. In some contexts, the results responded to and consolidated the significance of ethical leadership in CSR implementation and employees’ CSR engagement, which is similar to previous studies (e.g., Angus-Leppan et al., 2010; Freeman, 1988; Knippenberg and de Jong, 2010; Levine and Boaks, 2014; Nejati et al., 2020). Our study results support Choi et al. (2015), who found that the perceived ethical work environment encouraged supporters to have a moral work process by focusing on the well-being of the organization and reinforcing CSR through ethical leadership. In addition, our research included limited research on the CSR interface in emerging markets, such as the study by Pham and Tran (2020), which found that CEO integrity played an important role in driving the efficacy of CSR disclosure.

Second, the findings clarified the positive connection between CSR and firm reputation. We determined that companies implementing a CSR strategy enhanced their reputation both internally and externally, which demonstrates the practical benefits of CSR for businesses, as mentioned in previous studies (e.g., Lai et al., 2010; Stanaland et al., 2011; Stawiski et al., 2010; Worcester, 2009). Therefore, the CSR–firm reputation relationship is relevant to Vietnam, as Vietnamese customers expect companies to set standards accordingly while complying with legislation and regulatory systems to minimise the negative social and economic consequences (Huang et al., 2019).

The third hypothesis on the relationship between firm reputation and firm performance was determined to be positive in the findings. It was found that CSR activities' firm reputation positively affects firm performance in both financial and non-financial terms. By correctly implementing CSR, firms can convert their reputation into increased profit, talent and customers to establish a sustainable competitive advantage (Black et al., 2000; Sabate and Puente, 2003; Sundström et al., 2020). Finally, the analysis showed a correlation between CSR and firm performance. According to the findings and increased firm reputation, CSR has other direct effects on firm performance. Suppose the firm's reputation attracts more customers and talent; in that case, CSR impacts talent and customer retention by increasing satisfaction. Furthermore, implementing CSR could strengthen firms' operating productivity and effectiveness (Dhaliwal et al., 2011; Sharfman and Fernando, 2008; Zhou, 2016). Overall, the connection between CSR, firm reputation and firm performance, as explored in this study, will be a valuable reference for researchers who aim to study CSR in different contexts.

### 5.2 Practical implications

In addition to its theoretical implications, this study has several practical implications that offer interested parties an efficient, measurable CSR management tool. The findings show that ethical leadership plays an indispensable role in successfully implementing a CSR strategy. Corporate leaders must transform themselves into ethical leaders. To this end, leaders need to consider CSR as corporate social integration; therefore, they must develop a collaborative environment to remove the barriers to adopting CSR practices (Saha et al., 2020).

Second, the way that CSR creates a firm reputation should be differentiated from other forms of marketing. This means that before designing and implementing a CSR strategy, companies must clearly understand the theoretical concept of CSR and how the approach works. CSR in Vietnam still means voluntary philanthropy or charitable donations. The definition of ‘CSR as added value’, where CSR is one of the main business goals and part of the corporate strategy, has not been completely understood or adopted. For example, CSR in Vietnam tends to be viewed as a simplistic approach that only considers customers and the community and frequently ignores the commitment of companies to enhance employee welfare (Vuong et al., 2021). If businesses misunderstand the CSR concept, it will lead to improper actions and unfavourable outcomes. Additionally, CSR must be perceived by managers as an essential and indispensable component to be integrated into the business strategy of the company, including the consolidated cooperation of shareholders and top management. The proper distribution of resources should be regarded as an investment in a precious immaterial asset rather than solely a cost.

Third, because of the causal relationship between firm reputation and firm performance, companies will not achieve a sustainable goal and could weaken their competitive advantage if they implement a CSR strategy improperly; this problem is particularly relevant to large and multinational companies. Vinamilk, which is the largest Vietnamese dairy company, is a common example of gaining a competitive advantage due to a successful CSR strategy implementation. This company continually fulfils its product, environmental, capacity and social obligations. Although the company was in the business for half a century, it continues to gain and build profits because of its CSR practice and efforts.

Finally, the involvement of stakeholders in CSR implementation is necessary to enhance both firm reputation and firm performance. According to Slack et al. (2015), the sense of responsibility, enthusiasm and CSR-objective-engagement of the workforce is essential to deliver CSR initiatives and succeed with CSR implementation. Additionally, the affiliation between the firm and employees and the employees' self-interest contributes to the firm performance. This is noteworthy since employees are the only ones who can profoundly understand their working methods and conditions (DiMaggio and Powell, 1983). Accordingly, to attract attention and retain the stakeholders' involvement, the company's CSR strategy has to be simple, measurable and effective from the beginning. It is difficult to change and reform the mindset and working style of stakeholders and even more complicated and challenging if the stakeholders do not understand the meaning and efficiency of the company's CSR implementation. We suggest that the companies consider all their stakeholders while designing and implementing a suitable CSR strategy. If possible, the company should let the stakeholders contribute to the planning process, which is a straightforward approach to understanding their desires or constraints. Moreover, as mentioned above, it would be beneficial to start with small projects to test the reaction of the stakeholders, as this would remove obstacles and upgrade CSR activities.

### 5.3 Limitations and future research directions

We acknowledge that there are several limitations of this study. First, due to the various controversial theories and ideas related to the CSR concept, this study does not discuss or debate the correct method. Instead, it refers to theories to define all possible research areas. This approach is applied to all other multi-dimension concepts and norms that are used in this paper. We compensate for this through extensive research using official and reliable information sources and practical observance. Finally, the collected data from managers may contain subconscious biases, which could affect the results. This is because it could indicate the superficial perspective of the managers' position and not consider the other stakeholders.

Several opportunities derived from the above could be valuable suggestions for future studies of CSR. First, there are very few research papers that focus on Vietnamese case studies. Therefore, future studies could investigate other undiscovered or unpopular aspects of CSR or re-assess and expand this proposed research model to include an analysis of more practical case studies in Vietnam. Second, as the data sample is large in terms of the industry sector, this research model could be tested in a single industry sector that contributes to business practitioners in that area. Third, because the possible existence of psychological biases may occur in this paper, other researchers could test the hypotheses of this study from other stakeholders’ perspectives. Finally, the social and political environment has varied over time due to the development of the economy; therefore, it is crucial to undertake longitudinal research to gain more comprehensive information.

## Declarations

### Author contribution statement

Nguyen Thi Thao Nguyen: Analyzed and interpreted the data; Contributed reagents, materials, analysis tools or data; Wrote the paper.

Nguyen Phong Nguyen: Conceived and designed the experiments; Contributed reagents, materials, analysis tools or data; Wrote the paper.

Tu Thanh Hoai: Analyzed and interpreted the data; Wrote the paper.

### Funding statement

This work was supported by the University of Economics Ho Chi Minh City, Vietnam.

### Data availability statement

Data will be made available on request.

### Declaration of interests statement

The authors declare no conflict of interest.

### Additional information

No additional information is available for this paper.
